# Does Calcium Supplementation Negate Erythropoiesis With Endurance Training?

**DOI:** 10.1111/apha.70108

**Published:** 2025-09-26

**Authors:** Meihan Guo, David Montero

**Affiliations:** ^1^ Department of Medicine Beth Israel Deaconess Medical Center, Harvard Medical School Boston Massachusetts USA; ^2^ Faculty of Medicine, School of Public Health Hong Kong University Pokfulam Hong Kong; ^3^ Department of Medicine, School of Clinical Medicine Hong Kong University Pokfulam Hong Kong; ^4^ Libin Cardiovascular Institute of Alberta University of Calgary Calgary Canada

**Keywords:** endurance training‐induced erythropoiesis, hemoglobin mass, mineral supplementation, sex differences

Endurance training (ET) effectively enhances aerobic capacity in healthy humans by increasing circulating hemoglobin mass (Hb_mass_) [1]. We and others have observed that improved peak O_2_ consumption (VO_2peak_) after ET is reverted to pre‐training values following blood withdrawal to negate the ET‐induced gain in Hb_mass_ [2, 3]. Likewise, increases in Hb_mass_ and VO_2peak_ are determined by the ET dose [4]. Consequently, we are skeptical of large improvements in VO_2peak_ not primarily underlain by hematological adaptations, and expect that lifestyle interventions, including ET as the main stimuli, enhance VO_2peak_ along with erythropoiesis.

Dietary calcium supplementation is highly prevalent in developed countries. Around half of the US population, including 70% of older women, supplement their diets with calcium. Calcium supplementation is also used as a placebo in pharmacological trials, assuming its negligible effects on hematological and cardiovascular systems [5]. However, in the past century, animal studies demonstrated large alterations in hemoglobin (Hb) concentration with calcium supplementation [6, 7]. Recently, a study implementing 8 weeks of ET combined with calcium supplementation as a placebo did not increase Hb_mass_ but substantially enhanced VO_2peak_ (9% to 17%) in healthy women and men [5]. In that study, the modality of ET did not comprise typical (upright) exercises such as cycling or running, but rowing, which may entail central hemodynamic alterations negatively interacting with the endocrine regulation of erythropoiesis [8]. In fact, the rowing intervention induced isolated left atrial enlargement, commonly reflecting chronic pressure overload [8]. Therefore, the question remains whether typical ET combined with calcium supplementation (ET‐Ca) increases VO_2peak_ without eliciting hematological adaptations. Here, we tested the hypothesis that 8 weeks of upright cycling ET‐Ca enhances VO_2peak_ without concomitant increases in Hb_mass_ in healthy women and men. A control intervention for ET alone was not implemented since previous ET interventions in our laboratory, applying similar exercise stimuli for 6–8 weeks, resulted in proportional gains in Hb_mass_ and VO_2peak_ [4, 9].

Healthy men and women (*n* = 30, 43% ♀) matched by sex, age (38.5 ± 16.5 vs. 43.6 ± 14.5 years, *p* = 0.378), body mass index (BMI) (22.3 ± 3.2 vs. 21.7 ± 2.8 kg m^−2^, *p* = 0.302) and moderate‐to‐vigorous physical activity (5.2 ± 2.5 vs. 4.9 ± 3.7 h week^−1^, *p* = 0.811) were recruited via printed/online advertisements in the city of Hong Kong. All individuals were non‐obese (body mass index (BMI) < 30), normotensive, and non‐smokers. Inclusion criteria comprised healthy status according to clinical questionnaires and resting echocardiography/ECG screening, absence of current medical symptoms and medication, and no history of chronic disease. The study was approved by the Institutional Review Board of the University of Hong Kong/Hospital Authority West Cluster (UW 22‐025). Participants were allocated to 8 weeks of ET‐Ca. The ET program comprised 28 upright cycling ergometry exercise sessions (3–4 per week, every other day). All ET sessions had a fixed average intensity of 75% of peak heart rate (HR_peak_) for 50 min of duration. Calcium supplementation comprised orally ingested calcium carbonate tablets (500 mg, Shandong Yuwang Pharmaceutical). The tablets were ingested 4 h before each ET session. The measurement of hematological variables (Hb_mass_, red blood cell volume (RBCV), plasma volume (PV), blood volume (BV), erythropoietin (EPO)) and aerobic capacity followed established protocols in our laboratory, recently reported in detail [10, 11]. Statistical analyses (SPSS 26.0, IBM) included two‐way ANOVA with repeated measures with sex and time (pre, post) as between‐ and within‐subject factors, and their interaction.

ET‐Ca did not alter Hb_mass_ and RBCV in men (865 ± 194 vs. 868 ± 237 g, *p* = 0.878; 2657 ± 589 vs. 2665 ± 524 mL, *p* = 0.894) and women (550 ± 75 vs. 569 ± 103 g, *p* = 0.414; 1691 ± 230 vs. 1752 ± 317 mL, *p* = 0.407). PV was increased with ET‐Ca in women (3163 ± 380 vs. 3430 ± 574 mL, *p* = 0.027) but not in men (4065 ± 681 vs. 4187 ± 774 mL, *p* = 0.224). BV was not augmented with ET‐Ca in men (6722 ± 1176 vs. 6852 ± 1427 mL, *p* = 0.395) and women (4854 ± 542 vs. 5182 ± 842 mL, *p* = 0.083). Circulating EPO was not increased with ET‐Ca in men (20.1 ± 5.6 vs. 22.3 ± 10.7 mlU mL^−1^, *p* = 0.316) and women (17.7 ± 5.2 vs. 20.9 ± 4.9 mlU mL^−1^, *p* = 0.075). Interactions between sex and time were not observed (*p* ≥ 0.190). Figure 1 illustrates the effects of ET‐Ca on aerobic exercise capacity. ET‐Ca increased VO_2peak_ and peak power output (W_peak_) in men (*p* < 0.001) and women (*p* < 0.001). Interactions between sex and time were not observed (*p* ≥ 0.127).

**FIGURE 1:**
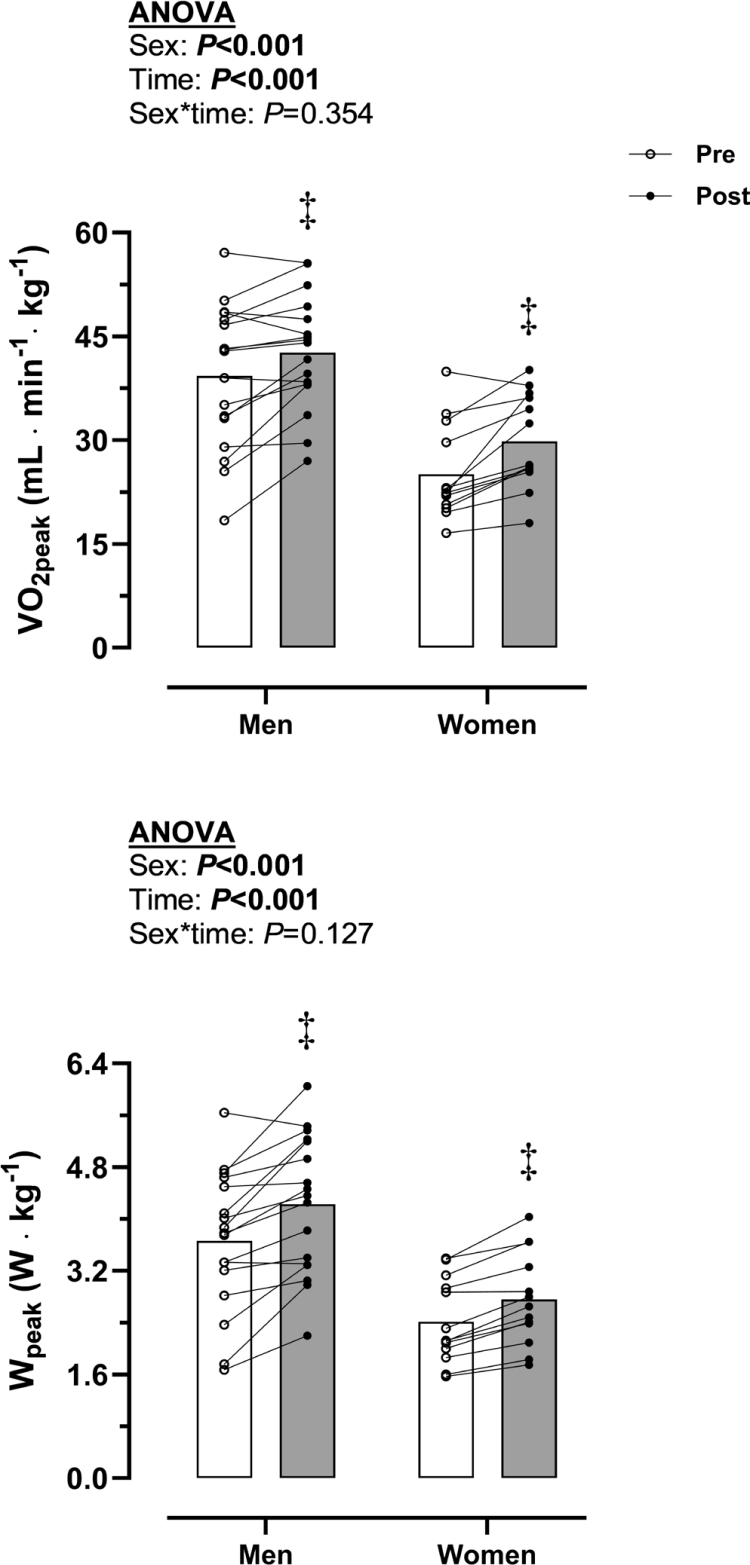
Effect of endurance training combined with calcium supplementation (ET‐Ca) on aerobic exercise capacity. Data in bars represent mean values. Significant *p* values (*p* < 0.05) for main factors in ANOVA (sex, time) and interaction are highlighted in bold. ^‡^ *p* < 0.05 between time points (“post” vs. “pre”) in each sex. The significance symbol (‡) is only illustrated above the “post” point to enhance visual clarity. VO_2peak_, peak O_2_ uptake; W_peak_, peak power output.

The results confirmed the tested hypothesis, but raise the question as to how calcium supplementation impairs ET‐induced erythropoiesis. Three quarters of a century ago, experiments on mice demonstrated marked (up to 46%) decrements in Hb concentration with calcium supplementation [6], and similar results were obtained on rats in the 1990s [7]. The addition of iron along with calcium supplementation partly prevented the reduction of blood O_2_ carrying capacity [6]. Nonetheless, the calcium‐induced (partial) blockade of iron absorption seems to be transient and cannot fully explain the reduction of erythropoiesis over several weeks in humans [12]. Alternatively, drugs that increase the concentration of calcium in blood (hypercalcemia) elicit drastic decreases in circulating EPO [13]. In this respect, we did not observe reductions in circulating EPO. As such, the underlying mechanisms remain elusive. Notwithstanding, our study unfolds the intriguing possibility that widely used performance‐enhancing drugs, that is, glucocorticoids, which lower the concentration of calcium in blood, might accelerate erythropoiesis [14].

ET enhances aerobic exercise capacity in healthy individuals without exception, provided the dose of endurance exercise is high enough [4]. In our study, the increment in VO_2peak_ concurred with the expected effects of moderate doses of ET in healthy individuals [4]. The increment in VO_2peak_ also matched that of the prior study implementing ET and calcium supplementation in healthy women and men [5]. Likewise, in both studies, ET‐Ca induced a mild increase in PV, herein reaching significance in women. However, we previously determined that isolated expansion of PV does not enhance VO_2peak_ in healthy women and men [15]. Accordingly, calcium supplementation does not blunt the effect of ET on VO_2peak_, but must alter its main underlying mechanism towards a non‐hematological one, yet to be discovered.

In conclusion, ET combined with calcium supplementation does not enhance the hematological determinants of aerobic exercise capacity in women and men. However, aerobic exercise capacity is improved with ET‐Ca in both sexes. Therefore, augmented circulating Hb_mass_ and BV expansion are not indispensable for moderate improvements in VO_2peak_ in healthy adults. The potential role of calcium in the regulation of exercise‐induced erythropoiesis opens new lines of investigation for clinical and performance‐enhancing aims.

## Conflicts of Interest

The authors declare no conflicts of interest.

## Data Availability

The data that support the findings of this study are available from the corresponding author upon reasonable request.
